# Neural Organoid Models as a Platform for Studying Disease Mechanisms in Amyotrophic Lateral Sclerosis

**DOI:** 10.1111/jnc.70513

**Published:** 2026-07-01

**Authors:** Kristel N. Eigenhuis, Roberto Montoro Ferrer, R. Jeroen Pasterkamp

**Affiliations:** ^1^ Department of Translational Neuroscience University Medical Center Utrecht Utrecht CG the Netherlands

## Abstract

Amyotrophic lateral sclerosis (ALS) is a fatal neurodegenerative disorder affecting upper and lower motor neurons leading to muscle wasting. However, structural and molecular abnormalities, including cortical thinning and TDP‐43 pathology, extend into frontal, parietal, and temporal areas, pointing to defects across broader cortical regions. The advent of human induced pluripotent stem cell (hiPSC) technology has enabled the generation of human‐specific brain cell types in vitro. Here, we provide an overview of the three‐dimensional (3D) hiPSC‐derived neural organoid platforms used to model cortical structures and to study cortical ALS‐associated phenotypes. We review which pathological hallmarks have been recapitulated in these organoids and discuss disease phenotypes reported to date. Further, we comprehensively cover different neural organoid models and experimental strategies, including patient‐derived hiPSC models and exogenous pathology induction, while addressing current technical challenges. Together, these advances position neural organoids as an emerging tool to study cell‐type‐specific and circuit‐level mechanisms related to cortical changes in ALS.

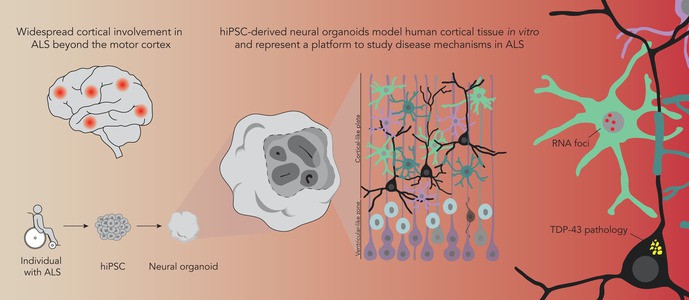

## Graphical abstract

Amyotrophic lateral sclerosis (ALS) involves widespread cortical pathology beyond the motor cortex. Human‐induced pluripotent stem cell‐derived neural organoids model cortical tissue in vitro and provide a physiologically relevant platform to study disease mechanisms in ALS.

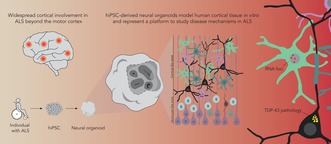

**Abbreviations**
AD: Alzheimer's disease
AGN: angiogenin
ALI‐COs: air‐liquid interface cortical organoids
ALS: amyotrophic lateral sclerosis
BDNF: brain‐derived neurotrophic factor
BMP: bone morphogenetic protein
*C9ORF72*
: chromosome 9 open reading frame 72
CEs: cryptic exons
CRISPR: clustered Regularly Interspaced Short Palindromic Repeats
DMN: diseased motor neuron
DPRs: dipeptide repeat proteins
EFG: epidermal growth factor
EIF2α: eukaryotic initiation factor 2 alpha
FGF2: fibroblast growth factor 2
FTD: frontotemporal dementia
*FUS*
: fused in sarcoma
GA: glycine–alanine
GFAP: glial fibrillary acidic protein
GoF: gain‐of‐function
GP: glycine–proline
GR: glycine–arginine
GRN: granulin
GWAS: genome‐wide association studies
hiPSC: human induced pluripotent stem cell
HM: homozygous mutant
HRE: hexanucleotide repeat expansions
ISS: International Space Station
LoF: loss‐of‐function
MAP 2: microtubule‐associated protein 2
MEA: multielectrode array
MRI: magnetic resonance imaging
mtSNVs: mitochondrial single nucleotide variants: Linker of Nucleoskeleton and Cytoskeleton LINC complex
NLS: nuclear localization signal
NMDA: N‐methyl‐D‐aspartate
NMJ: neuromuscular junctions
NT3: neurotrophin 3
PABP1: polyA‐binding protein 1
PD: Parkinson's disease
pTDP‐43: phosphorylated TDP‐43
RA: retinoic acid
RAN: repeat associated non‐AUG
*SOD1*
: superoxide dismutase 1
*TARDBP*
: TAR DNA‐binding protein
*TBK1*
: TANK‐Binding Kinase 1
TDP‐43: TAR DNA‐binding protein 43
TGF‐ß: transforming Growth Factor‐beta
TUNEL: terminal deoxynucleotidyl transferase dUTP nick end labeling
UPR: unfolded protein response
XPO1: Exportin 1

## 1 Introduction

Amyotrophic lateral sclerosis (ALS) is an adult‐onset and rapidly progressive neurodegenerative disorder with a lifetime risk of 1:400 (Xu et al. 2020). ALS is defined by the progressive loss of lower motor neurons in the spinal cord and brainstem, along with degeneration of upper motor neurons in the motor cortex. This leads to muscle weakness and atrophy, ultimately resulting in death due to respiratory failure. Following symptom onset, median survival is 3–5 years, and treatment options remain limited (Van Es et al. 2017).

In approximately 10% of ALS patients, there is a clear family history of the disease (Goutman et al. 2022; Al‐Chalabi 2013). Currently, more than 30 associated genes have been identified in these familial cases (Van Rheenen et al. 2021). Although the remaining 90% of cases are considered sporadic, ALS is a complex disease for which heritability is estimated at 40%–50% (Van Rheenen et al. 2021). The most common ALS‐linked genes, chromosome 9 open reading frame 72 (*C9ORF72*), superoxide dismutase 1 (*SOD1*), TAR DNA‐binding protein (*TARDBP*), and fused in sarcoma (*FUS*), harbor pathogenic variants that account for approximately 60% of familial cases and about 10% of sporadic ALS (Akçimen et al. 2023; Hardiman et al. 2017). To date, genome‐wide association studies (GWAS) have identified several genome‐wide significant loci that explain only a small fraction of genetic susceptibility to ALS (Nakamura et al. 2020; Nicolas et al. 2018; Van Rheenen et al. 2016). Some of these loci (such as *C9ORF72* and TANK‐Binding Kinase 1 (*TBK1*)) also carry rare large‐effect variants (Dejesus‐Hernandez et al. 2011; Cirulli et al. 2015), whereas the rare‐variant contribution of the remaining loci is unknown (Van Rheenen et al. 2021).

ALS is not only genetically complex, but also a clinically heterogeneous disease that shares pathobiological features with frontotemporal dementia (FTD). Whereas 50% of ALS patients manifest only motor symptoms, up to 50% develop cognitive or behavioral changes (Van Es et al. 2017). Detailed population‐based phenotyping data show that about 13% of patients have concomitant behavioral‐variant FTD (Elamin et al. 2013; Phukan et al. 2012). The discovery of non‐coding hexanucleotide repeat expansions (HRE) in *C9ORF72* as the major genetic cause of ALS and FTD proves that these disorders are extremes on the phenotypic spectrum of a single disease (Van Rheenen et al. 2021).

The pathogenesis of ALS remains incompletely understood, yet gene defects that characterize specific genetic subtypes converge onto a common set of cellular and molecular pathways. These include abnormalities in RNA metabolism, toxic protein aggregation, mitochondrial dysfunction, proteostasis and autophagy failure, cytoskeletal proteins, oxidative stress, and neuroinflammation (Van Es et al. 2017; Goutman et al. 2022; Faller et al. 2025). Linking pathogenic processes to specific genetic mutations has helped to show which molecular mechanisms primarily drive ALS. Defects in the other pathways may arise secondarily but still contribute to disease progression.

### 1.1 Overview of the Most Common ALS‐Linked Genes

#### 1.1.1 TDP‐43

While *TARDBP* mutations are infrequent, accounting for ~3%–5% of familial ALS and < 1% of sporadic cases, the presence of cytoplasmic aggregates containing the protein TAR DNA‐binding protein 43 (TDP‐43) in nearly all ALS patients identifies TDP‐43 as the central pathological hallmark of the disease (Sreedharan et al. 2008; Neumann et al. 2006). TDP‐43 is an RNA/DNA binding protein that normally mainly localizes to the nucleus, with essential functions in transcription, RNA processing, and splicing. To date, more than 80 dominant disease‐associated variants have been identified. The majority of these mutations cluster in the intrinsically disordered C‐terminal domain of TDP‐43, which contains aggregation‐prone motifs and plays a central role in pathological inclusion formation. A smaller number of mutations affect the N‐terminal domain required for TDP‐43 oligomerization and RNA processing. TDP‐43 pathology involves nuclear clearance leading to a loss‐of‐function (LoF), and cytoplasmic mislocalization followed by hyperphosphorylation and cleavage. Cleaved TDP‐43 can exhibit toxic gain‐of‐function (GoF) properties via increased hydrophobicity, sequestration of essential cellular components, and generation of oxidative species.

According to neuropathological staging (Brettschneider et al. 2013; Braak et al. 2013), phosphorylated TDP‐43 (pTDP‐43) inclusions in ALS first develop in projection neurons of the motor cortex and somatomotor neurons in the brainstem and spinal cord. At subsequent stages, pathology develops in the prefrontal cortex, reticular formation, brainstem nuclei, striatum, and basal ganglia. At late stages, TDP‐43 pathology extensively progresses into the temporal lobe, entorhinal cortex, and in the hippocampal and dentate fascia (Jo et al. 2020). Current classification largely focuses on dystrophic neurites and neuronal cytoplasmic inclusions, often overlooking non‐neuronal, such as glial, pathology. Nevertheless, TDP‐43–positive inclusions are also commonly observed in glial cells, particularly oligodendrocytes and astrocytes.

Accumulating evidence suggests that prion‐like spreading of pathological TDP‐43 aggregates may be involved in the progression of ALS. Misfolded TDP‐43 can be propagated from cell‐to‐cell in a seed‐dependent and self‐templating manner, similar to aberrant protein aggregates in other neurodegenerative diseases such as Alzheimer's disease (AD) and Parkinson's disease (PD) (Jo et al. 2020).

#### 1.1.2 C9ORF72‐HRE

Expansion of the intronic hexanucleotide (G_4_C_2_) repeat in *C9ORF72* is the most common genetic cause of ALS, observed in 40% of familial cases and 10% of sporadic cases (Dejesus‐Hernandez et al. 2011; Renton et al. 2011). C9ORF72 is broadly expressed across human tissues, with particularly high levels in the brain and spinal cord. Its localization is primarily cytoplasmic, with punctate staining in neurites indicating a synaptic role. C9ORF72 is detected in synaptosome preparations from mouse brains and co‐localizes with synaptic markers in mouse brain sections and human induced pluripotent stem cell (hiPSC)‐derived motor neurons (Atkinson et al. 2015; Ferguson et al. 2024). In neurons, C9ORF72 is proposed to regulate actin dynamics and endosomal recycling of GluR1 at the synapse. Studies using tagged, transfected, or Clustered Regularly Interspaced Short Palindromic Repeats (CRISPR)‐modified C9ORF72 in cell lines or hiPSC‐derived neurons have further revealed co‐localization of the protein with various organelles, including the Golgi apparatus, stress granules, mitochondria, and particularly components of the endolysosomal pathway (Smeyers et al. 2021).

Wild‐type alleles of the *C9ORF72* gene carry fewer than 30 hexanucleotide repeats in the first intronic region, whereas pathogenic alleles have been reported to range between 700 and 1600 repeats (Renton et al. 2011). This HRE causes cellular dysfunction via (1) formation of intra‐nuclear RNA foci that sequester RNA‐binding proteins, (2) repeat associated non‐AUG (RAN) translation of G_4_C_2_ repeat RNA into dipeptide repeat proteins (DPRs), and (3) hypermethylation of the G_4_C_2_ repeats leading to C9ORF72 haploinsufficiency (Vatsavayai et al. 2019; Balendra and Isaacs 2018). RNA foci are prominent in motor neurons in both the motor cortex and spinal cord but can also be identified in non‐neuronal cells such as glia (astrocytes, oligodendrocytes, microglia). An initial study (Dejesus‐Hernandez et al. 2011) reported no differences in C9ORF72 protein levels in lymphoblasts or brain lysates from FTD or ALS patients carrying the HRE compared to non‐carriers. However, subsequent studies using improved antibodies have shown a 25%–50% reduction of C9ORF72 in the frontal, occipital, motor, and temporal cortices of affected patients.

#### 1.1.3 SOD1

SOD1 is a highly conserved, ubiquitously expressed copper–zinc superoxide dismutase that protects cells from oxidative stress by catalyzing the conversion of superoxide radicals into oxygen and hydrogen peroxide. Although primarily cytosolic, SOD1 also localizes to the nucleus. Pathogenic variants in *SOD1* account for approximately 1%–6% of ALS cases worldwide, with notable geographic variation (Rosen et al. 1993). SOD1‐associated ALS is neuropathologically defined by cytoplasmic hyaline conglomerate inclusions in degenerating motor neurons that co‐label for SOD1 and neurofilaments, and that are unique to this genetic form of ALS. Beyond its antioxidant role, SOD1 has additional functions in regulating gene expression and mRNA stability. However, the contribution of these roles to ALS pathogenesis remains unclear (Bosco et al. 2010).

Many studies indicate that mutant SOD1 causes a toxic GoF effect that drives pathogenesis. However, evidence for loss of SOD1's enzymatic activity in ALS disease also exists (Benatar and Munch Andersen 2025). Mutant SOD1 appears to induce neuronal death via multiple pathways, including excitotoxicity, oxidative stress, mitochondrial dysfunction, impaired axonal transport, and toxic effects from surrounding glial cells. Misfolded wild‐type SOD1 has also been implicated in sporadic ALS, indicating that both wild‐type and mutant forms can independently contribute to disease. These insights helped position SOD1‐related ALS as one of the earliest neurological conditions targeted for molecular therapies aimed at reducing misfolded SOD1 (Van Es et al. 2017; Salzinger et al. 2024).

#### 1.1.4 FUS

FUS is a 526‐amino‐acid RNA‐binding protein with a complex domain structure, including an N‐terminal ‘prion‐like’ low‐complexity domain, glycine‐rich region, RNA recognition motif, zinc finger, and C‐terminal nuclear localization signal (NLS), and is highly conserved and ubiquitously expressed. It functions in gene regulation, RNA processing, and is a component of heterogeneous ribonucleoprotein complexes, RNA granules, and stress granules (Yulei Shang 2016). Although predominantly nuclear in neurons and glia, FUS shuttles to the cytoplasm where it contributes to mRNA transport and local protein synthesis at synapses and interacts with other proteins, including TDP‐43 (Nomura et al. 2014).

Disease‐causing variants of *FUS* are clustered in the NLS‐region of the gene, and they account for 4% of familial and 1% of sporadic ALS. Upon disruption of its nuclear localization, FUS‐immunoreactive cytoplasmic inclusions with variable morphologies are formed in upper and lower motor neurons, and cortical regions such as the basal ganglia, thalamus, and midbrain (Lattante et al. 2013; Moens et al. 2025). In a subset of FUS‐ALS cases, FUS‐positive inclusions can also be detected in the cytoplasm of oligodendroglia and are referred to as glial cytoplasmic inclusions. FUS also forms inclusions in around 5% of FTD patients. However, distinct pathogenic processes have been suggested to underlie the two diseases (Moens et al. 2025).

### 1.2 Widespread Cortical Involvement in ALS: Far Beyond the Motor Cortex

**FIGURE 1:**
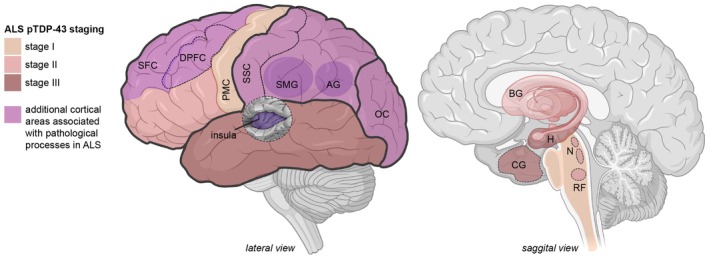
Widespread TDP‐43 pathology in multiple cortical areas in ALS. Progression of pathological phospho(p)‐TDP43 inclusions throughout (sub)cortical areas in ALS at different stages as described by Braak et al. (2013) and Brettschneider et al. (2013). Purple areas represent additional cortical areas that have been linked to pathological processes in ALS as well, such as cortical thinning, spreading of TDP‐43 pathology, and gene‐expression changes. The frontal‐, temporal‐, parietal‐, and occipital lobe are outlined in black. AG, angular gyrus; BG, basal ganglia; DPFC, dorsal prefrontal cortex; EC, enthorhinal cortex; H, hippocampal & dentate fascia; N, nuclei of the brainstem; OC, occipital cortex; PMC, primary motor cortex; RF, reticular formation; SC, spinal cord; SCC, somatosensory cortex; SFC, superior frontal cortex; SMG, supramarginal gyrus.

Increasing imaging, neuropathological, and clinical evidence shows that ALS is not restricted to motor neurons, and that multiple cortical and subcortical regions beyond the primary cortex show structural and molecular pathology (Thorns et al. 2013; Takeuchi et al. 2016; Tan et al. 2023; Shen et al. 2023; Dadar et al. 2020). These areas show characteristic abnormalities such as TDP‐43 pathology, cortical thinning, disrupted connectivity, and selective neuronal vulnerability, particularly in regions rich in specialized projection neurons (Figure 1).

Magnetic resonance imaging (MRI) morphometry studies show that ALS patients exhibit cortical thinning not only in motor areas but in widespread non‐motor cortical areas. Reported regions are the pre‐ and postcentral gyri, the superior and inferior parietal lobule, angular and supramarginal gyrus, insula, superior frontal, temporal, and occipital regions. Importantly, cortical thickness is related to clinical severity (Thorns et al. 2013), and consistent with spreading of TDP‐43 pathology across the cortex (Tan et al. 2023; Shen et al. 2023; Dadar et al. 2020). Post‐mortem studies also show that in sporadic ALS, pTDP‐43 aggregates often extend beyond the motor system into frontal, temporal, and other neocortical regions. Their distribution is heterogeneous across patients and correlates with cognitive impairment, supporting possible clinico‐pathological subtypes of ALS (Takeuchi et al. 2016). A recent large‐scale single‐nucleus RNA sequencing study reported convergent, cell‐type–specific gene‐expression changes in both the motor cortex and dorsolateral prefrontal cortex across sporadic ALS, familial ALS, and FTD cases. Disease signatures as well as non‐cell autonomous pathological mechanisms were identified in neuronal and non‐neuronal cell types in multiple cortical regions. These observations highlight the fact that ALS‐related vulnerability extends beyond traditional motor regions (Sebastian Pineda et al. 2024).

This widespread cortical involvement helps explain the cognitive and behavioral changes commonly observed across the ALS–FTD spectrum and indicates that ALS progresses through large‐scale cortical networks rather than being confined to motor pathways. Whereas neuropathological, neuroimaging, and clinical studies can demonstrate *where* pathology manifests, they often capture late‐stage consequences of disease. This highlights the importance of experimental models that more accurately recapitulate human cortical biology and circuit‐level dynamics. Mechanistic insights into *how* ALS pathology emerges, which cell types initiate it, or which interactions drive progression are needed. Ultimately, these insights are crucial to enable the development of targeted interventions to effectively treat the disease.

Studies using two‐dimensional (2D) hiPSC‐derived neuronal models have provided important insights into ALS pathogenesis, particularly regarding neuron degeneration, protein aggregation, and cellular stress responses. However, these systems lack the spatial organization and circuit‐level interactions needed to model the widespread cortical involvement observed in ALS patients. For this reason, our review focuses specifically on neural organoids, which offer a 3D, multilayered, and developmentally informed human model capable of capturing the cellular diversity and network dynamics that underlie ALS pathogenesis. This review is primarily focused on cortical organoids as a model of upper cortical neurons and cortical pathology in ALS and FTD. However, because cortical organoid applications in ALS remain relatively limited, we also discuss spinal cord and neuromuscular organoids as complementary models that capture other components of motor circuit disease.

## 2 Neural Organoid Models

Human iPSCs have reshaped neuroscience research by providing a means to generate human‐specific brain cell types in vitro (Takahashi and Yamanaka 2006). hiPSCs can be differentiated into either 2D cultures or 3D organoid models. 2D cultures, including monocultures and co‐cultures, are useful for investigating cell‐autonomous properties and intercellular interactions. 3D organoid models take advantage of the cell‐intrinsic developmental programs to generate complex structures that recapitulate aspects of the cellular diversity and tissue architecture of human neurodevelopment (Velasco et al. 2020; Faravelli et al. 2025).

**FIGURE 2:**
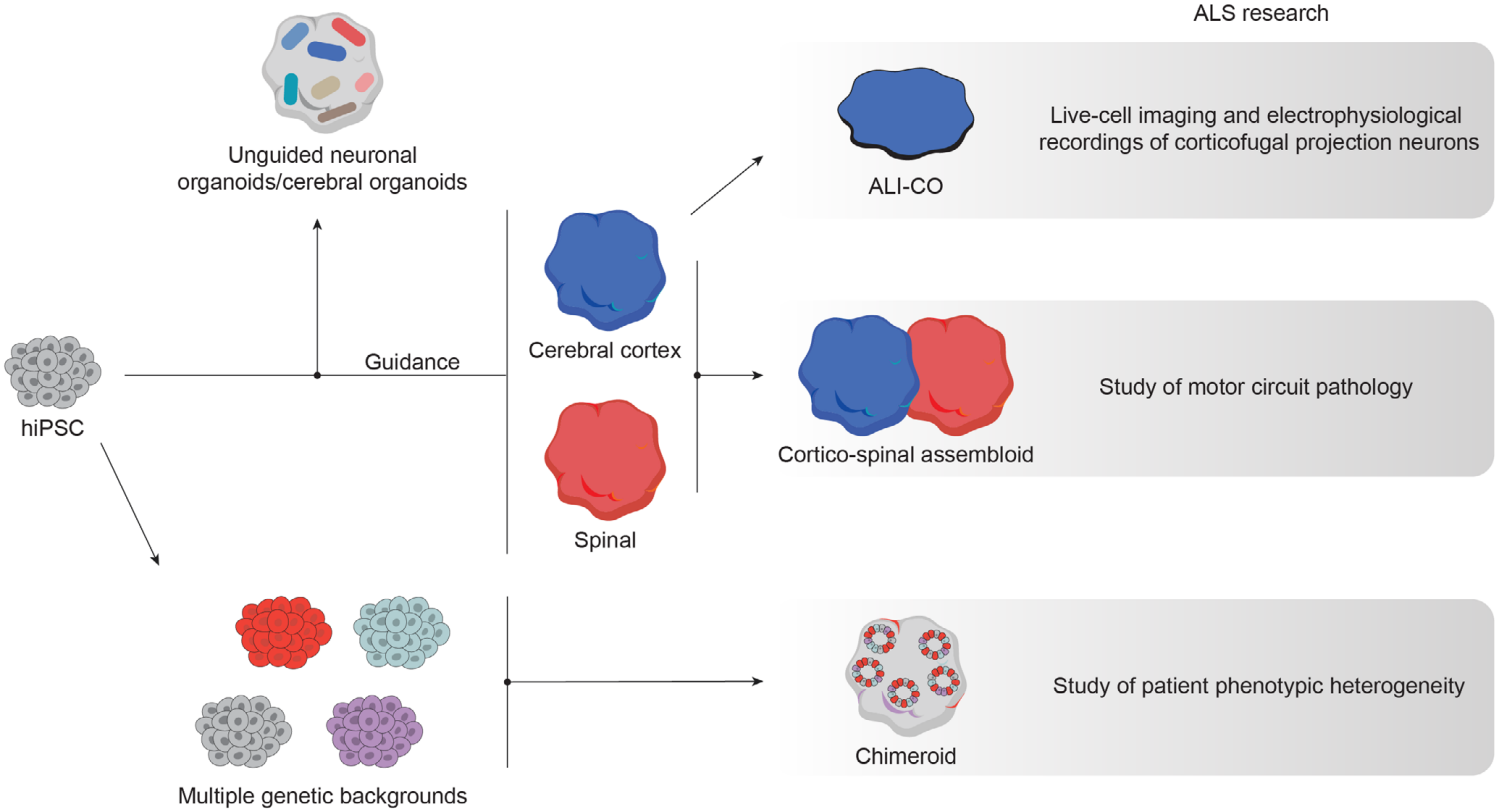
Neural organoid models and ALS research. hiPSC can differentiate into organoids without or with molecular guidance, generating unguided neural organoids or regionalized organoids, respectively. Regionalized organoids can be sliced and cultured in an air‐liquid interface system and can also be combined into assembloids. Additionally, hiPSC from multiple genetic background can be differentiated into chimeroids. Gray boxes at the right indicate the potential use of each model in ALS research.

hiPSC‐derived neural organoids can be generated through guided or unguided approaches (Figure 2) (Pașca et al. 2022). Guided differentiation generates regionalized neural organoids that self‐pattern into a specific region of the nervous system, such as in cortical organoids. In contrast, unguided differentiation supports the generation of heterogeneous organoids containing neural populations that reflect multiple nervous system regions, also referred to as cerebral organoids (Takahashi and Yamanaka 2006). However, this acquired regional heterogeneity is generally unpredictable, offering limited control over regional specification or reproducibility. During guided differentiation protocols, external patterning cues are added to enhance control over cell fate and regional identity, improving organoid generation reproducibility (Paşca et al. 2015; Velasco et al. 2019). Cortical organoids are guided neural organoids that recapitulate key hallmarks of human corticogenesis, including the self‐organization of radial glia‐like cells lining ventricular structures and the sequential generation of deep‐ and upper‐layer cortical neurons. In addition, cortical organoids recapitulate aspects of gliogenesis and can contain astrocytes and oligodendrocytes. Moreover, microglia progenitors can be transplanted into cortical organoids. Free‐floating cortical organoids are the most widely used approach (Mototsugu Eiraku et al. 2008).

Several research groups have developed protocols with ever evolving strategies for generating cortical organoids, with protocols from the Arlotta and Pasça laboratories representing two of the most widely adopted approaches (Vieira De Sá et al. 2021). Both protocols rely on guided differentiation of hiPSCs toward dorsal forebrain progenitors, generating organoids that model human corticogenesis in vitro. The Arlotta protocol guides hiPSCs into 3D aggregates under dual pathway inhibition, targeting WNT and TGF‐ß signaling, and transitions the organoids into spinner‐flask bioreactors to promote growth, self‐organization, and nutrient access. Similarly, the Pasça protocol uses concurrent inhibition of Bone Morphogenetic Protein (BMP) and Transforming Growth Factor‐beta (TGF‐ß) signaling pathways (dual SMAD inhibition) during early neural induction to establish dorsal forebrain identity, followed by careful modulation of growth conditions, including the addition of epidermal growth factor (EFG) and fibroblast growth factor 2 (FGF2) followed by neurotrophin 3 (NT3) and brain‐derived neurotrophic factor (BDNF) to support maturation of cortical progenitors and neurons. Both approaches reproducibly generate free‐floating organoids with dorsal cortical identity, capturing the temporal dynamics of corticogenesis and producing neuronal and glial populations that closely resemble those of the human fetal cortex. These widely used protocols provide a robust foundation for studying human cortical development and modeling disease‐relevant processes in vitro.

Free‐floating cortical organoid protocols provide a robust system for capturing the temporal dynamics that define corticogenesis. Over extended periods of culture, free‐floating cortical organoids progressively increase in cellular diversity, neuronal subtype maturation, and dendritic and axonal processes. Following in vivo timelines, cortical organoids develop ventricle‐like structures composed of radial glia‐like cells surrounding a lumen within a month in culture. These progenitors give rise to deep‐layer cortical neurons within 2 months and upper‐layer cortical neurons within 3 months, at which point radial glia‐like progenitors switch to generating glia cells. Importantly, cortical organoids can achieve postnatal‐like stages after 250–300 days of differentiation (D250‐D300), with transcriptional, epigenetic, and physiological hallmarks of maturation that mirror in vivo development, including N‐methyl‐D‐aspartate (NMDA) receptor and histone deacetylase complex subunit switching (Gordon et al. 2021). Extending these timelines further, human cortical organoids have been cultured for up to 5 years, during which excitatory neurons remain viable and transcriptionally age in a cell type‐specific manner (Faravelli et al. 2025). Whole‐genome methylation profiling revealed that the predicted epigenetic age closely tracks time in culture, paralleling in vivo brain aging. Collectively, these studies highlight that free‐floating cortical organoids not only recapitulate early corticogenesis but also provide an unprecedented window into postnatal‐like maturation of human brain development.

Building on free‐floating cortical organoid cultures, organotypic slice‐inspired methods section organoids and culture them as tissue slices, giving rise to air‐liquid interface cortical organoids (ALI‐COs) (Giandomenico et al. 2019; Marta Cañizares Luna et al. 2025). The air‐liquid interface improves tissue oxygenation, supports long‐term neuronal survival, and promotes axonal outgrowth. Importantly, ALI‐COs preserve the characteristic laminar organization of cortical progenitors and neurons. This approach facilitates experimental access and is well‐suited for live‐cell imaging and electrophysiological neuronal recordings among other techniques. ALI‐COs and cortical organoids provide a platform to study ALS pathology in disease‐relevant neurons, such as deep‐layer corticofugal projection neurons and early cortical network dysfunction.

Chimeroids further extend the organoid protocol repertoire by introducing a modular, reaggregation‐based method (Anton Bolanos et al. 2024). In this approach, hiPSC are first aggregated and guided toward a dorsal forebrain lineage. The resulting progenitors are then dissociated and reassembled, producing organoids with improved uniformity in cortical identity across organoids. A key advantage of chimeroids is their modular design, which allows the combination of progenitors from different individuals into a single organoid. This offers a powerful platform for studying how interindividual genetic variation shapes cortical development, cellular interactions, and disease susceptibility. In ALS research, this advantage represents an opportunity to better understand patient‐specific variability. By stabilizing early patterning while preserving the capacity for self‐organization, chimeroids provide a system for modeling genetically complex neurodevelopmental and neurodegenerative disorders.

Beyond the cortex, spinal cord organoids are guided neural organoids that recapitulate human spinal cord development and are composed of spinal cord cells, including dorsal sensory neurons, ventral motor neurons, as well as astrocytes and oligodendrocytes (James et al. 2021). Spinal cord organoid protocols are based on patterning cues that guide hiPSCs toward caudal neuroectoderm, such as retinoic acid (RA). Spinal cord organoids provide a system to study lower motor neuron vulnerability in ALS.

Complementing advances in increasingly reproducible neural organoid systems, assembloids extend organoid‐based modeling by enabling the reconstruction of multi‐compartment neural interactions and circuit‐level organization in vitro. Assembloids are generated through the fusion and functional integration of two or more regionally specified organoids, allowing cells to migrate, extend axons, and form functional connections across organoids. This modular framework enables the integration of region‐specific neural organoids to reconstruct neural circuits in vitro, such as cortico‐thalamic, cortico‐striatal, or cortico‐spinal assemblies (Miura et al. 2020; Andersen et al. 2020; Ji‐Il Kim et al. 2024). By combining relevant brain regions, assembloids allow the study of fundamental neurodevelopmental processes that are inaccessible in isolated organoids, including interneuron migration from ventral to dorsal forebrain compartments and long‐range axon guidance (Miura et al. 2020; Birey et al. 2017; Bagley et al. 2017). More recently, this approach has been extended to model closed‐loop circuits through the development of loop assembloids, in which cortical, striatal, thalamic, and midbrain organoids are integrated into a cortico–striatal–thalamic–cortical circuit (Miura et al. 2020; Ji‐Il Kim et al. 2024). Loop assembloids recapitulate key features of reciprocal circuit connectivity and coordinated network activity, providing direct experimental access to the assembly and dysfunction of human loop circuits implicated in neurodevelopmental and neuropsychiatric disorders. For ALS, assembloids could be used to assess the implications of different nervous system regions.

Together, these different organoid approaches provide complementary entry points into human cortical development. Each approach is best suited to address specific biological questions, ranging from early progenitor development and neuronal differentiation to synaptic maturation, molecular pathogenesis, or long‐range connectivity. In the following sections, we focus on cortical organoids as a disease model of upper cortical neurons for FTD and ALS. Since the application of cortical organoids to ALS research remains limited, we provide a comprehensive perspective by also examining other neural organoid models, including cerebral and neuromuscular organoids. In our literature search, we included all studies in which organoids were used to investigate ALS. We first review ALS‐associated pathological hallmarks that have been recapitulated in cortical organoids and then discuss how these systems have been used to model disrupted cellular and molecular processes linked to ALS.

## 3 Pathological Hallmarks of ALS in Neural Organoid Models

Here, we summarize pathological hallmarks of ALS that have been recapitulated in neural organoid models. We define pathological hallmarks as molecular or cellular lesions that correspond to disease‐associated features observed in ALS patient tissue, including protein and RNA inclusions, altered localization of disease‐defining proteins, and markers of neurodegeneration or genomic instability. Importantly, several of these hallmarks emerge constitutively in ALS‐linked genetic backgrounds, whereas others require cellular stress or experimental induction. See Table 1 for an overview.

**TABLE 1:** Pathological hallmarks of ALS that have been recapitulated in neural organoids.

Pathological hallmark	Organoid type and time in culture	Genetic background	Constitutive or induced	Additional	Ref
*Dipeptide repeats (DPR)*					
Poly(GA)	Cerebral organoid ALI‐Cos (D150)	C9ORF72‐HRE	Constitutive	↑ phospho‐EIF2α/EIF2α protein ratio ↑SG marker PABP1 ↑ total p62 signal	Szebényi et al. (2021)
Poly(GA) & poly(GP)	Cerebral organoids (D90)	C9ORF72‐HRE	Constitutive	Reduced C9ORF72 expression in C9ORF72‐HRE organoids	Van Der Geest et al. (2024)
Poly(GA)	Cerebral organoids (D60)	Sporadic ALS/FTD (no mutation identified)	Exogenous seeding of C9ORF72‐ALS spinal cord extracts	Colocalization with pTDP‐43 aggregation	(Tamaki et al. 2023)
Poly(GA)	Neuromuscular organoids—in neurons & astroglia (D100)	C9ORF72‐HRE	Constitutive	↑p62 puncta in neurons & astroglia	Gao et al. (2024)
*TDP‐43 pathology*					
↑phosphorylated TDP‐43 ↑ extranuclear TDP‐43 localization	3D brain organoid‐like structures (1 month)	Granulin (GRN)^−/−^	Constitutive		De Majo et al. (2023)
↑phosphorylated TDP‐43 aggregates ↑ nuclear TDP‐43 mislocalization	Cerebral organoids (D60)	Sporadic ALS/FTD (no mutation identified)	Exogenous seeding of C9ORF72‐ALS spinal cord extracts	↑ apoptosis (cleaved caspase 3, TUNEL) ↑ double‐strand DNA breaks (γH2AX foci)	Tamaki et al. (2023)
↑ nuclear TDP‐43 mislocalization	Spinal cord organoids—motor neurons (D120)	C9ORF72‐HRE	Constitutive		Sirtori et al. (2024)
↑phosphorylated TDP‐43 aggregates ↑ nuclear TDP‐43 mislocalization	Forebrain organoids (D107)	TDP‐43 ^K181E −/−^	Constitutive	pTDP‐43 co‐localization with cleaved caspase 3	Qi Zhang et al. (2025)
*Cryptic exon inclusion*					
*STMN2* CE inclusion	3D brain organoid‐like structures (1 month)	Granulin (GRN)^−/−^			De Majo et al. (2023)
*UNC13A* CE inclusion *POLDIP3* altered splicing	Cortical organoids (D45)	Control and C9ORF72‐HRE	Sodium arsenite chronic exposure	Altered p62 distribution	Casiraghi et al. (2025)
*RNA foci*					
RNA foci	Cerebral organoids (D90)	C9ORF72‐HRE	Constitutive	at D90	Van Der Geest et al. (2024)
RNA foci	Cerebral organoids with innately developed microglia (D60‐70)	C9ORF72‐HRE	Constitutive	RNA foci in neurons and subset of microglia, at D64	Ljubikj et al. (2025)

Abbreviations: CE, cryptic exon; D, days of differentiation; GA, guanine‐alanine; GR, guanine‐proline; pTDP‐43, phosphorylated TDP‐43; SG, stress granule.

### 3.1 Protein Accumulation and Impaired Proteostasis

Aberrant protein accumulation and impaired proteostasis are prominent pathological features of ALS and have been recapitulated most consistently in C9ORF72‐HRE‐associated cortical organoid models. Multiple studies using cortical or cerebral organoid derivatives generated from C9ORF72‐HRE hiPSCs report the accumulation of dipeptide repeat proteins, most prominently poly(GA), accompanied by alterations in proteostasis markers such as p62. In an ALI‐CO model, Szebenyi et al. (Szebényi et al. 2021) observed abundant accumulation of poly(GA) in C9ORF72‐ALS organoids compared to (isogenic) control organoids. This accumulation was accompanied by an increase in the phospho‐eukaryotic initiation factor 2 alpha (EIF2α)/EIF2α protein ratio, high levels of the stress granule marker poly(A)‐binding protein 1 (PABP1), and of p62. Notably, the increased p62 signal was enriched in astrocytic (glial fibrillary acidic protein, GFAP+) over neuronal (microtubule‐associated protein 2, MAP2+) cell populations. These findings suggest that DPR pathology in organoids is not restricted to neurons and may involve glial populations, consistent with emerging evidence from patient tissue. Importantly, pharmacological unfolded protein response (UPR) inhibition using a GSK compound reduced both UPR activation and poly(GA) accumulation, indicating that proteostasis‐related pathological inclusions in organoids are amenable to experimental modulation.

Alterations in p62 distribution have also been reported in other organoid studies. Casiraghi et al. (Casiraghi et al. 2025) described altered intracellular localization of p62 following induction of oxidative stress by sodium arsenite treatment, with accumulation observed in both C9ORF72‐HRE and control hiPSC‐derived cortical organoids compared to untreated conditions. While not specific to a genetic ALS background, these findings support the notion that cortical organoids can model disease‐relevant disturbances in proteostasis and inclusion‐associated markers under defined conditions.

DPR pathology has also been detected in cerebral organoid models. Van Der Geest et al. (2024) reported that C9ORF72‐HRE (809–1175 repeats) causes reduced C9ORF72 expression at developmental stages (D45) and the emergence of glycine–alanine (GA) and glycine–proline (GP) DPRs at more mature stages (D90) in patient‐derived neural organoids.

Evidence for DPR‐driven pathology has also been demonstrated using exogenous seeding approaches. In cerebral organoids derived from sporadic ALS–FTD patient hiPSCs (with no mutation identified), Tamaki et al. (2023) showed that injection of spinal cord extracts from C9ORF72‐ALS patients led to the appearance of poly glycine–arginine (GR) repeat proteins, whereas extracts from non‐C9ORF72 ALS cases did not. In these organoids, poly(GR) inclusions colocalized with phosphorylated TDP‐43 aggregates, suggesting that DPR pathology may propagate and induce secondary protein lesions in cortical organoids.

More recently, neuromuscular and cortical organoid models have reinforced the robustness of DPR pathology and its cell‐type specificity. Gao et al. (2024) demonstrated that poly(GA) puncta were significantly elevated in neurons and astrocytes of C9ORF72‐ALS‐derived neuromuscular organoids, while skeletal muscle cells showed little to no accumulation. Notably, poly(GA) inclusions were more pronounced in astrocytes than in neurons, again highlighting glial involvement in DPR pathology. In the same model, p62 puncta were increased in both astroglia and neurons at later stages, consistent with disturbed autophagic clearance. Acute and prolonged treatment with the UPR inhibitor GSK2606414 significantly reduced poly(GA) levels and partially normalized p62 accumulation. This further supports the utility of organoid systems for modeling both pathological inclusions and their pharmacological modulation.

Collectively, these studies demonstrate that cortical and cerebral organoid models robustly recapitulate DPR accumulation and proteostasis‐related inclusion pathology characteristic of C9ORF72‐ALS.

### 3.2 TDP‐43 Pathology and Related Neurodegenerative Cellular Damage

Pathological alterations of TDP‐43, i.e., nuclear depletion, cytoplasmic mislocalization, and hyperphosphorylation, constitute a defining neuropathological hallmark of ALS. Despite their central role in disease, these features have been proven challenging to recapitulate in human cortical organoid models under endogenous conditions. However, recent advances in the field have enabled the emergence of TDP‐43–related pathology in cortical organoids and closely related 3D human neural systems.

Several studies report elevated levels of pTDP‐43 and increased extranuclear localization in cerebral organoid models. De Majo et al. (2023) demonstrated significantly higher protein levels of pTDP‐43 in granulin‐deficient (GRN^−^/^−^) cerebral organoid‐like structures composed of mature induced astrocytes and neurons, accompanied by increased extranuclear localization of TDP‐43. Although GRN is classically linked to FTD, it can be used within the ALS‐FTD spectrum as a tractable model to study shared TDP‐43 associated pathomechanisms. These findings indicate that progranulin LoF alone is sufficient to drive TDP‐43 pathology in a 3D cortical context.

TDP‐43 pathology has also been observed in the earlier mentioned organoid model of Tamaki et al. (2023), which used exposure to pathological protein seeds. Post‐mortem spinal cord protein extracts from sporadic ALS patients were injected into cerebral organoids derived from sporadic ALS‐FTD or control hiPSCs. Extracts from ALS patients induced the formation of pTDP‐43 pathology that progressively increased over time, becoming evident at 2‐, 4‐, and 8‐weeks post‐injection. In contrast, control organoids injected with ALS extracts exhibited only minor pTDP‐43 pathology at late time points. Eight weeks after injection, ALS‐FTD cerebral organoids displayed abundant cytoplasmic pTDP‐43 aggregates in neurons and GFAP‐positive astrocytes, accompanied by reduced nuclear TDP‐43. In control organoids, TDP‐43 expression remained nuclear in all cell types. These findings demonstrate that ALS patient–derived protein extracts can seed and propagate TDP‐43 pathology in cerebral organoids and that this pathology extends to non‐neuronal cell types. However, the specific extract components responsible for these effects remain unclear, and future studies could further fractionate the patient‐derived material to define the active component.

Importantly, extract‐induced TDP‐43 pathology was associated with neurodegeneration‐related cellular damage, i.e., increased apoptosis (terminal deoxynucleotidyl transferase dUTP nick end labeling (TUNEL), and cleaved caspase‐3) and elevated γH2AX foci indicative of double‐strand DNA breaks. Both apoptotic markers and γH2AX colocalized with pTDP‐43 aggregates, linking seeded TDP‐43 pathology to cellular degeneration in this model.

Although not strictly cortical organoids, related 3D neural models provide complementary evidence for TDP‐43 pathology. Sirtori et al. (2024) reported TDP‐43 mislocalization in a subset of motor neurons within C9ORF72‐ALS spinal cord organoids, indicating that TDP‐43 pathology can emerge in organoid‐based systems in the absence of direct TARDBP mutations. Similarly, Sharma et al. (2024) used motor neuron–containing organoids in which TDP‐43 was overexpressed in neurons to generate a “diseased motor neuron” (DMN) model. Compared to healthy organoids, DMN organoids cultured on Earth exhibited increased levels of TDP‐43 (De Majo et al. 2023). Exposure of DMN organoids to spaceflight conditions aboard the International Space Station (ISS) further exacerbated TDP‐43 pathology, suggesting that microgravity and space environment may accelerate neurodegeneration and amplify TDP‐43–associated pathological features in 3D human neural cultures (De Majo et al. 2023).

Recent studies show that direct modeling of *TARDBP* mutations in cortical organoids enables the recapitulation of TDP‐43 pathology. Using human forebrain organoids derived from hiPSCs carrying the ALS‐associated TDP‐43‐K181E mutation, Qi Zhang et al. (2025) reported a clear genotype‐dependent increase in pTDP‐43. pTDP‐43 was minimal in control organoids, moderately increased in heterozygous mutant organoids, and most pronounced in homozygous mutant (HM) organoids. Total TDP‐43 protein levels were comparable between wild‐type and mutant organoids, indicating that the observed pathology reflects altered localization and post‐translational modification rather than increased expression. Importantly, HM organoids exhibited a significant fraction of cells depleted of nuclear TDP‐43, recapitulating a key neuropathological feature of ALS. To further assess subcellular localization, organoid cells were dissociated and cultured as a monolayer, revealing cytoplasmic accumulation of pTDP‐43 that in some cases colocalized with cleaved caspase‐3, linking TDP‐43 pathology to neurodegeneration‐associated damage. In a related study using the same organoid model, Chin et al. (2025) investigated whether TDP‐43 mislocalization could be pharmacologically modulated. Treatment with KPT‐276, an inhibitor of the nuclear export receptor exportin 1 (XPO1), significantly reduced cytoplasmic pTDP‐43–positive puncta in TDP‐43‐K181E mutant organoids. Notably, KPT‐276 treatment did not alter total TDP‐43 protein levels in either mutant or control organoids, indicating selective modulation of pathological TDP‐43 localization rather than global expression.

Taken together, these studies demonstrate that cortical organoids and neural organoid models can recapitulate multiple facets of TDP‐43 pathology, including involvement of astrocytes. While the extent of mislocalization and aggregation varies across models, the consistent emergence of these lesions highlights the potential of organoid systems for modeling early and intermediate stages of TDP‐43 pathology, as well as therapeutic interventions relevant to ALS.

### 3.3 RNA‐Related Pathological Hallmarks

#### 3.3.1 Cryptic Exon Inclusion and Loss of TDP‐43 Splicing Function

TDP‐43 functions as a key splicing regulator that represses the inclusion of cryptic exons (CEs) in neuronal transcripts. Loss of this splicing control is considered a central component of TDP‐43 LoF pathogenesis. Under conditions of TDP‐43 proteinopathy, aberrant CE inclusion in genes such as *UNC13A, POLDIP3*, and *STMN2* introduces premature termination or polyadenylation signals, resulting in reduced expression of proteins essential for neuronal homeostasis (Klim et al. 2019; Ma et al. 2022; Brown et al. 2022). Disruption of TDP‐43–mediated RNA splicing has been modeled in cortical organoids through both genetic and stress‐induced paradigms. De Majo et al. (2023) reported robust inclusion of a CE in *STMN2* in granulin‐deficient brain organoid‐like structures, consistent with loss of TDP‐43 nuclear function. Importantly, selective loss of progranulin in astrocytes was sufficient to induce both TDP‐43 pathology and cryptic *STMN2* splicing defects in organoids containing wild‐type neurons. This demonstrates a non–cell‐autonomous mechanism by which diseased astrocytes drive RNA dysregulation in neighboring neurons. Partial rescue of these phenotypes upon treatment with exogenous full‐length progranulin further supports a causal link between progranulin deficiency and TDP‐43–dependent splicing dysfunction.

Stress‐induced loss of TDP‐43 splicing activity has also been captured in C9ORF72‐ALS and control cortical organoids. Casiraghi et al. (2025) showed that chronic oxidative stress induced by sodium arsenite exposure triggered significant inclusion of a CE in *UNC13A* and skipping of exon 3 in *POLDIP3* in control organoids, revealing that environmental stress alone is sufficient to compromise TDP‐43 splicing fidelity. In C9ORF72‐HRE organoids, sodium arsenite treatment similarly promoted *UNC13A* CE inclusion, with a trend toward altered *POLDIP3* splicing. Notably, pharmacological enhancement of autophagy via rapamycin significantly reduced these splicing defects in stressed C9ORF72‐HRE organoids, but not in controls, linking RNA dysregulation to impaired proteostatic clearance.

#### 3.3.2 RNA Foci Formation in C9ORF72 Organoid Models

Pathogenic RNA foci arising from *C9ORF72‐HRE* transcripts have been consistently observed across multiple organoid systems. In cerebral organoids, Van Der Geest et al. (2024) detected intranuclear RNA foci at later stages of maturation, coinciding temporally with the emergence of DPR pathology. Cell‐type–resolved analyses further revealed that RNA foci are not restricted to neurons. Ljubikj et al. (2025) identified RNA foci in both neurons and a subset of microglia (~11%) within C9 ALS/FTD organoids, indicating that repeat‐associated RNA pathology extends to immune cell populations in organoids.

In contrast, Gao et al. (2024) reported nuclear RNA foci selectively in neurons of C9ORF72‐ALS neuromuscular organoids, with no detectable foci in astrocytes or skeletal muscle cells. Together, these findings highlight substantial cell‐type specificity in RNA foci formation across different 3D human models, likely reflecting differences in cellular vulnerability, maturation state, repeat expression length, or transcript levels. Collectively, the detection of RNA foci in several cell types in different organoid models highlights the robustness of this hallmark in organoids.

## 4 ALS Disease Phenotypes in Human Neural Organoid Models

**FIGURE 3:**
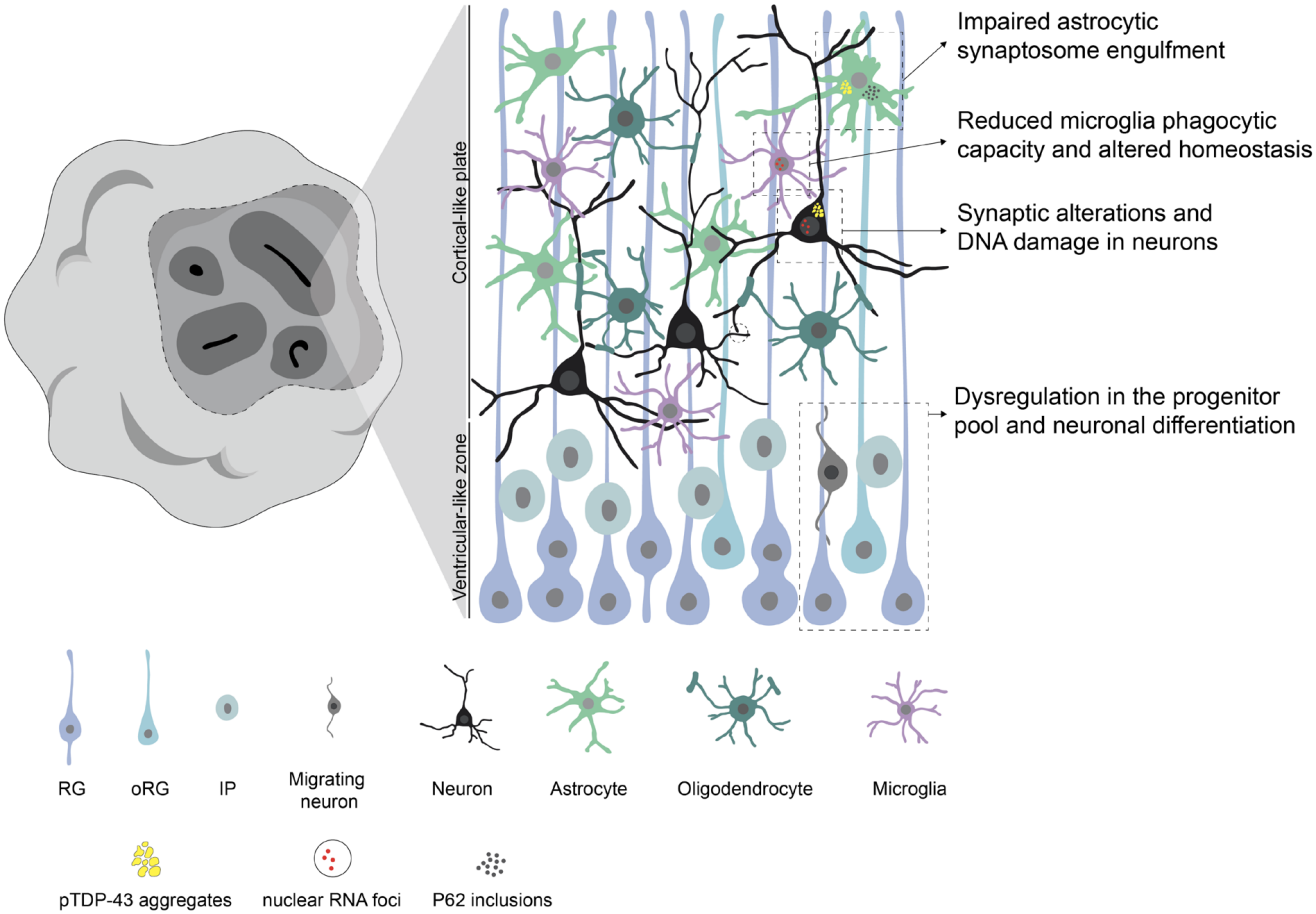
ALS‐associated pathogenic mechanisms studied in neural organoids Left: Schematic representation of a neural organoid with a dashed‐line cutout indicating a view into the interior, revealing ventricle‐like structures. Right: Magnified view of one ventricle‐like structure highlighting key cell types and tissue organization, including ventricular zone‐like and cortical plate‐like structures. Different neural cell types are depicted, including radial glia cells (RG), outer radial glia (oRG), intermediate progenitors (IP), migrating newborn neurons, cortical neurons, astrocytes, oligodendrocytes, and microglia. Arrows indicate ALS‐associated pathogenic mechanisms studied using organoid studies, mapped onto specific cell types.

Beyond the recapitulation of canonical ALS pathological hallmarks, human neural organoids have enabled the identification of downstream pathogenic phenotypes that inform how disease‐associated mechanisms translate into cellular dysfunction and circuit impairment. Both pathological hallmarks and disease phenotypes of ALS that have been modeled in human neural organoids are summarized in Figure 3.

### 4.1 Progenitor Proliferation and Neuronal Differentiation Dysregulation

Organoid systems enable the study of how ALS‐associated mutations impact human neurodevelopment. In cortical and spinal cord organoids, FUS LoF impacts progenitor proliferation. FUS knockout cortical organoids displayed increased neuronal progenitor proliferation, while spinal cord organoids showed reduced progenitor proliferation (Zou et al. 2022). In both systems, disrupted progenitor proliferation was accompanied by alterations in neuronal differentiation. Mechanistically, FUS is known to bind to the mRNA of the neurotrophin‐3 receptor NTRK3, a Trk tyrosine kinase receptor whose expression increases during organoid differentiation. Accordingly, in FUS‐deficient organoids, NTRK3 levels were increased across both regional identities.

A disruption of progenitor dynamics was also evident in ALS‐FTD‐related angiogenin (AGN) mutant cerebral organoids, which exhibited disruptions in early neurodevelopment characterized by the persistence of disorganized ventricular‐like rosettes at later stages (Ferguson et al. 2024). The progenitor pool defects were accompanied by increased numbers of SOX2+ neural progenitors, and in dividing cells. While there was no change in overall neuronal numbers, AGN mutant organoids displayed increased late‐born cortical neurons positive for CTIP2 and CUX1 and a reduction in early‐born TBR1+ neurons.

In C9ORF72‐HRE cerebral organoids, neurodevelopmental abnormalities initially appeared as accelerated organoid growth accompanied by a broader distribution of Ki67+ dividing neuronal progenitors within ventricular‐like zones (Van Der Geest et al. 2024). However, this early proliferative phase was followed by impaired maturation, as C9ORF72‐HRE organoids were significantly smaller at later stages and contained fewer deep‐layer cortical neurons.

Together, these findings suggest that early disruptions in progenitor pool proliferation propagate into long‐term deficits in neuronal composition, indicating that developmental dysregulation may prime ALS cortical neurons for increased vulnerability at later stages.

### 4.2 Synaptic Connectivity Alterations

As organoids mature in vitro, neurons establish functional neuronal networks that enable the interrogation of ALS‐associated synaptic phenotypes. In a preprint, transcriptomic analysis of TDP‐43‐K181E forebrain organoids revealed early gene expression changes in excitatory neurons. In addition, single‐cell RNA sequencing showed strong overlap between genes upregulated in mutant organoids and those elevated in postmortem brain samples of dementia patients with confirmed TDP‐43 pathology (Qi Zhang et al. 2025). In contrast, although excitatory neurons showed reduced expression of key developmental and connectivity‐associated genes, including SATB2 and TENM2, the synaptic connectivity pathways found downregulated in patient tissue were unaltered in organoids. In contrast, organoid‐like structures formed by a combination of hiPSC‐derived neurons and astrocytes revealed alterations in neuronal communication. In this system, granulin LoF was associated with an increase in both pre‐ and post‐synaptic puncta, suggesting a disruption in synaptic connectivity (De Majo et al. 2023).

Synaptic alterations were also evident in C9ORF72‐HRE cerebral organoids. These organoids showed reduced expression of glutamatergic synaptic genes across neuronal populations, together with changes in presynaptic organization. This included fewer and smaller synaptophysin‐positive puncta and reduced presynaptic protein intensity, while postsynaptic SHANK2 puncta were largely preserved (Van Der Geest et al. 2024). These structural changes were associated with functional deficits, as electrophysiological recordings revealed depolarized resting membrane potential and a reduced frequency of spontaneous excitatory postsynaptic currents. This suggests reduced synaptic connectivity and neuronal activity.

Collectively, these studies identify disruption of neuronal communication as a pathogenic phenotype in ALS organoids, suggesting that early molecular and cellular perturbations can translate into lasting connectivity deficits in ALS organoid models.

### 4.3 DNA Damage and Nuclear Integrity Defects

Human organoids have revealed that ALS pathogenesis is accompanied by compromised genome integrity in a cell‐type specific manner. In cerebral organoids exposed to spinal cord extracts from ALS patients, TDP‐43 pathology correlated with the accumulation of double‐strand DNA breaks, indicating that pathogenic proteins may initiate genome instability and trigger cell death (Tamaki et al. 2023). In C9ORF72‐HRE ALI‐CO, neurons, but not astrocytes, exhibited DNA damage that could be partially rescued by pharmacological modulation of cellular stress pathways, suggesting that neuronal genome instability arises downstream of broader cellular stress responses (Szebényi et al. 2021). Moreover, single‐cell mitochondrial genome profiling in C9ORF72‐HRE ALI‐CO further identified increased accumulation of pathogenic mitochondrial single nucleotide variants (mtSNVs) in astrocytes relative to neurons, revealing cell‐type specific vulnerabilities in DNA maintenance and mitochondrial function (Nie et al. 2025). Thus, hiPSC‐derived organoids reveal how genetic mutations and pathogenic proteins converge on genome instability as a cell‐type specific phenotype of ALS pathogenesis.

Beyond direct DNA lesions, organoids have helped to uncover structural defects in nuclear architecture that may predispose ALS neurons to genome instability. In C9ORF72‐HRE spinal cord organoids, motor neurons displayed disruption of the Linker of Nucleoskeleton and Cytoskeleton (LINC) complex, characterized by mislocalization and reduced abundance of its main subunits, SUN1 and SUN2 (Sirtori et al. 2024). Importantly, analysis of post‐mortem cortical neurons revealed that disruption of SUN1 correlated with altered nuclear morphology in the absence of TDP‐43 mislocalization. This suggests that LINC complex defects can arise independently of canonical TDP‐43 pathology. Together with the findings in spinal cord organoids, these data position LINC complex disruption as a structural nuclear phenotype that may contribute to ALS pathogenesis upstream of, or parallel to, TDP‐43 aggregation.

Collectively, organoid studies helped to identify genome instability as a cell‐type‐specific and mechanistically relevant feature of ALS pathogenesis.

### 4.4 Glia Dysfunction and Neuron–Glia Signaling Deficits

The cellular complexity of organoid models provides a unique platform to investigate how glia dysfunction contributes to ALS pathogenesis. In C9ORF72‐ALS cerebral organoids containing endogenous microglia, transcriptional profiling revealed a widespread downregulation of gene networks related to microglia homeostasis, lysosomal function, phagocytosis, and antigen presentation, as well as a deregulation of key microglia transcriptional regulators such as PU.1 (Ljubikj et al. 2025). These molecular alterations were accompanied by functional impairments, including reduced phagocytic capacity and lysosomal defects in C9ORF72‐ALS‐derived organoid microglia, along with a reduction in synaptic marker density at the organoid level. Together, these findings argue against a hyperinflammatory state but hint at microglia‐mediated synaptic changes. Consistent with this, C9ORF72‐ALS organoids displayed attenuated cytokine and chemokine release upon inflammatory challenge. This indicates a blunted or maladaptive immune response rather than excessive neuroinflammation.

Astrocyte dysfunction has also been studied in GRN−/− organoid‐like structures, wherein granulin LoF induced downregulation of astrocytic phagocytosis pathways and functionally impaired synaptosome engulfment (De Majo et al. 2023). These alterations point to disrupted astrocyte–neuron signaling and highlight non‐cell‐autonomous aspects of disease pathogenesis.

Organoid models have also revealed aberrant intercellular signaling linked to neuronal stress. In a preprint using TDP‐43‐K181E mutant forebrain organoids, excitatory neurons showed increased autocrine and paracrine signaling via ligand‐receptor pairs such as PTN‐PTPRZ1, SLIT1‐ROBO1, and PTPRS‐NTRK3 (Qi Zhang et al. 2025). A similar implication of NTRK3 was observed in FUS‐deficient organoids, suggesting convergent stress‐adaptive signaling across ALS genotypes (Zou et al. 2022).

Complementing genetic organoid models, environmental challenges have also been leveraged to study neuroinflammation. Exposure of cortical and motor neuron organoid‐like structures to microgravity during spaceflight induced a broad increase in neurodegeneration‐associated biomarkers, including ALS‐relevant markers TDP‐43 and kallikrein‐related peptidase 6 (KLK6) in both healthy and TDP‐43 overexpressed organoids (Sharma et al. 2024). While this platform does not resolve cell‐type‐specific inflammatory responses, treatment with nanoligomer candidates screened for downregulation of inflammatory markers mitigated the disease‐associated biomarker signature induced by spaceflight.

Together, these studies demonstrate that organoids can uncover glial pathogenesis. Rather than modeling neuroinflammation as an end‐stage response, organoids suggest that glial dysfunction may act as an early and mechanistically active contributor to neuronal impairment in ALS.

### 4.5 Neuromuscular Connectivity Defects

Human neuromuscular organoids provide a platform to model the interface between spinal cord motor neurons, interneurons, glia, and skeletal muscle. In ALS hiPSC‐derived neuromuscular organoids (carrying mutations in *SOD1*, *PFN1*, or *TDP‐43*), neuromuscular junctions (NMJ) displayed structural and functional defects, including reduced innervation in *SOD1* and *PFN1* mutant lines and a reduction in NMJ area size in TDP‐43 mutant organoids (Pereira et al. 2021).

In addition, C9ORF72‐ALS patient‐derived neuromuscular organoids displayed progressive peripheral neuromuscular dysfunction. Functional deficits emerged by D50, with C9ORF72‐ALS organoids presenting a reduction in contraction frequency despite a preserved NMJ structure. By D100, this phenotype had progressed to reduced skeletal muscle contraction associated with a reduced ratio of integrated NMJs, defined as Bassoon‐positive presynaptic puncta aligned with neuron‐projected acetylcholine receptors and SMI32‐positive motor neurons, and with a loss of S100+ glia cells at the post‐synapse (Gao et al. 2025). These changes were associated with disturbed autophagy in neurons and astrocytes and reduced calcium signaling. Short‐term pharmacological treatment with the UPR inhibitor GSK2606414 transiently increased skeletal muscle contraction in C9ORF72‐ALS organoids. Prolonged treatment failed to improve contractility and was associated with alterations in NMJ and nicotinic acetylcholine receptor integrity, highlighting the utility of neuromuscular organoids for phenotypic drug testing.

## 5 Technical Innovation, Challenges & Future Directions for Modeling ALS in Neural Organoids

Despite their rapid development in the field, neural organoids have thus far only modeled a subset of ALS pathogenic mechanisms. Most studies have focused on C9ORF72‐HRE and TDP‐43‐associated mechanisms and pathologies. Interestingly, thus far changes associated with C9ORF72‐ALS appear more robust and consistent in neural organoid models. RNA foci and DPR accumulation frequently emerge in C9ORF72‐HRE organoids without the need for external stressors or artificial induction. This spontaneous manifestation suggests that cortical organoids are particularly well suited to capture intrinsic, cell‐autonomous disease mechanisms driven by C9ORF72‐HRE. In contrast, endogenous TDP‐43 pathology remains notably difficult to model. In most sporadic ALS or TDP‐43‐ALS mutant organoid systems, TDP‐43 mislocalization and aggregation do not arise spontaneously unless highly aggressive mutations are used (Qi Zhang et al. 2025) or pathology is induced by external agents such as stress, seeding, or overexpression of proteins (Casiraghi et al. 2025; Tamaki et al. 2023). Rather than reflecting a failure of the organoid platform, this difference likely reflects fundamental biological differences between ALS subtypes and pathologies, suggesting that TDP‐43 pathology may depend more strongly on extrinsic triggers, age‐related processes, or specific cellular contexts not yet fully captured in younger cortical organoids. Importantly, neuronal maturation and biological aging are distinct processes, and recent work demonstrates that aging alone can drive TDP‐43 mislocalization (Kevin Rhine et al. 2025), highlighting the need to incorporate aging paradigms beyond long‐term culture into neural organoid models.

These observations highlight an important difference between genetic and externally induced models of ALS pathogenesis. Genetic models, in which disease‐associated mutations are present from early developmental stages onward, are particularly informative for studying endogenous gene expression changes, early pathogenic programs, and cell‐intrinsic vulnerabilities. Conversely, induced pathology models, relying on external stimuli, may be more appropriate for interrogating aggregation dynamics, pathology propagation, or (pathway‐specific) downstream toxic effects. Importantly, neither approach is inherently superior; rather, each addresses distinct mechanistic questions. Thus, while no single neural organoid model currently captures all ALS disease hallmarks, these models are highly valuable for investigating distinct mechanisms in ALS. This conclusion also derives from the fact that the field of organoid research for ALS is relatively recent and that most models remain incompletely characterized. For example, C9ORF72‐ALS studies have largely focused on C9ORF72‐associated hallmarks (DPRs, RNA foci, reduced C9ORF72) but have not extensively characterized other hallmarks such as TDP‐43 pathology or its downstream consequences, such as CE inclusion. Therefore, further characterization of the various genetic and induced organoid models will also increase their value and ability to model ALS more broadly.

Also, the further technological development of organoid models will increase their value for ALS‐related studies. Cortical organoids do not inherently generate all cortical cell types that are present in the cortex and affected by ALS. These organoids do generate various glial cell types and layer V‐like glutamatergic projection neurons, including subsets that transcriptionally resemble corticofugal or corticospinal (pyramidal tract‐like) neurons (He et al. 2024; Uzquiano et al. 2022). However, oligodendrocytes are scarce in this organoid system and should be a focus of future model development. The neurons generated in cortical organoids are not as mature and morphologically specialized as the corticospinal motor neurons found in vivo. This limitation persists despite the long culture time of organoid protocols, often extending past half a year of culture. Neuronal long‐range axonal projections, full electrophysiological maturation, and precise areal specification are limited, largely due to the absence of appropriate extrinsic cues, long‐distance targets, and often lack of key cell types like microglia or the absence of a vasculature system. As a result, these neurons are best described as corticofugal‐like, rather than corticospinal neurons.

To address these limitations, the development of more complex models such as assembloid systems will be essential. Cortico‐spinal and cortico‐spinal‐muscular assembloids will have the potential to investigate how cortical pathology influences downstream motor neuron degeneration and neuromuscular dysfunction and would enable the study of long‐range connectivity and synaptic integration. Current assembloid systems remain technically demanding and are often heterogeneous. However, developments in the field make it realistic to expect more reproducible and physiologically relevant models of ALS network changes in the near future. The earlier described protocol for multidonor human brain chimeroids that was recently developed could be such a promising approach to overcome variability in neural organoid models. As neural progenitors from multiple donors are co‐developed within a single organoid, this ensures a highly reproducible system that could provide a powerful platform for studying ALS‐relevant cellular phenotypes and inter‐individual variability.

Another focus for future cortical organoid studies of ALS should be on the integration of an aging component. Cortical organoids currently predominantly model early developmental stages rather than the aged neuronal states at which neurodegenerative diseases like ALS typically manifest. In neurons, processes such as the accumulation of aggregated proteins and dysregulation of oxidative phosphorylation heavily depend on neuronal aging. This could explain why TDP‐43 pathology is often not arising constitutively in current organoid models. Recent findings (Kevin Rhine et al. 2025) show that aging alone functionally destabilizes the neuronal stress response and TDP‐43‐mediated splicing and that aging‐linked deterioration of RNA biology is a key driver of poor resiliency in aged neurons. An aging component could possibly be introduced via transdifferentiation methods that result in alternative (older) cellular starting points for generating organoids. Further, the addition of compounds that induce aging could be considered.

In addition to more complex cultures, such as assembloids or chimeroids, technologies are being applied to cortical organoid models that increase their utility for ALS studies. For example, cortical organoids are well suited for integration with techniques such as multielectrode array (MEA) recordings, calcium imaging, and single‐cell or spatial transcriptomics, allowing high‐resolution functional and molecular analyses. These opportunities make cortical organoids promising platforms for therapeutic screening and personalized medicine using patient‐derived cells. Several studies have demonstrated that pharmacological interventions in cortical organoids can attenuate or reverse ALS‐associated pathogenesis (Szebényi et al. 2021; Casiraghi et al. 2025; Gao et al. 2024; Chin et al. 2025). Indeed, organoids represent an unprecedented tool for personalized medicine. In particular, chimeroids have the potential to serve as high throughput pharmacological screening platforms, as a single organoid can contain cells from multiple donors, capturing the phenotypic heterogeneity observed in ALS and enabling the assessment of individual responses to specific drug or drug combinations.

In summary, neural organoids represent a powerful approach for modeling distinct pathogenic and pathological mechanisms in ALS. They provide 3D platforms that can be derived from patient cells and that model complex cellular interactions also found in human brain tissue. Although the field of ALS‐related organoid research is relatively young, the first studies have provided important insights and show that different aspects of the ALS disease process can be faithfully modeled in vitro. It is expected that the number of genetic mutations and ALS pathways that are being studied using neural organoids will increase in the coming years and that technological advances will help to make these models more robust and broadly applicable. Increasing neuronal maturation and aging‐associated features, and application of more complex platforms will develop cortical organoids into an even more robust system for modeling ALS and other neurodegenerative diseases. Through continued technical innovation and integrative modeling approaches, cortical organoids can provide highly relevant insights into disease mechanisms and offer a platform for discovering novel therapeutic opportunities.

## Author Contributions

**Kristel N. Eigenhuis:** writing – original draft. **Roberto Montoro Ferrer:** writing – original draft. **R. Jeroen Pasterkamp:** funding acquisition, writing – review and editing, supervision.

## Funding

This work was supported by Stichting ALS Nederland, GoALS.

## Conflicts of Interest

The authors declare no conflicts of interest.

## Data Availability

Data sharing not applicable to this article as no datasets were generated or analysed during the current study.
